# National policy responses to maintain essential health services during the COVID-19 pandemic

**DOI:** 10.2471/BLT.21.286852

**Published:** 2021-12-02

**Authors:** Nikki Gurley, Elan Ebeling, Ashley Bennett, Jean-Jacques Kayembe Kashondo, Victoria Ayano Ogawa, Clemence Couteau, Claire Felten, Naakesh Gomanie, Pauline Irungu, Katharine D Shelley, Jessica C Shearer

**Affiliations:** a Center of Digital and Data Excellence, PATH, Seattle, United States of America (USA).; b Primary Health Care, PATH, Seattle, USA.; c Health Technology, Meta, Seattle, USA.; d Communicable Disease and Immunization, King County Public Health, Seattle, USA.; e Conversa Health, Seattle, USA.; f Advocacy and Public Policy, PATH, Washington, DC, USA.; g London School of Economics and Political Science, London, England.; h Advocacy and Public Policy, PATH, Nairobi, Kenya.; i Primary Health Care, PATH, 455 Massachusetts Avenue NW, Suite 1000, Washington, DC 20001, USA.

Essential health services – including services for human immunodeficiency virus (HIV) infection and/or acquired immunodeficiency syndrome (AIDS), tuberculosis, malaria, routine immunization, noncommunicable diseases, nutrition and reproductive, maternal, newborn, child and adolescent health – are foundational to primary health care and vital for protecting population health. The coronavirus disease 2019 (COVID-19) pandemic disrupted the delivery of essential health services in most countries, with ongoing and differing disruptions as the COVID-19 pandemic continues. A survey conducted by the World Health Organization (WHO) between May and July 2020 found that 90% (94/105) of countries responding had reported a disruption to such services, with lower- and middle-income countries reporting generally greater disruptions.^1^ In early 2021, 94% (127/135) of surveyed countries reported some disruption in the previous 3 months.^2^

Disruptions pose a threat to health outcomes, with low- and middle-income countries facing a disproportionate burden. For example, researchers estimate that excess deaths in low- and middle-income countries due to suspension of health services has the potential to erase decades of improvements in child and maternal mortality.^3^^,^^4^ Vulnerable populations, including women, children, internally displaced people and migrants, people with disabilities and people living in poverty, are the most affected by the COVID-19 pandemic and its secondary effects (such as effects on the economy, health and education).^2^ Disruption to health services risks widening existing inequities and leaving vulnerable populations even further behind.

## Policies to maintain health services 

National governments and international agencies acknowledged risks to continuity of care early in the COVID-19 pandemic and began developing policies (which we define to include policies, norms, guidelines and strategies) to maintain or adapt the delivery of essential health services. WHO published interim guidance for maintaining these services during the COVID-19 pandemic on 25 March 2020, releasing final guidance on 1 June 2020.^5^ Other global technical agencies, including but not limited to the Joint United Nations Programme on HIV/AIDS, Centers for Disease Control and Prevention, RBM Partnership to End Malaria, United Nations Population Fund and other WHO departments released guidance on specific technical areas, including for HIV/AIDS, malaria and immunization. 

To track policy development at a national level, we launched the COVID-19 Essential Health Services Policy Tracker^6^ in collaboration with WHO’s Maternal, Adolescent, Child Health and Ageing department with funding from the Bill & Melinda Gates Foundation. The tracker identified and analysed 198 policy documents across 53 countries through September 2020. Our analysis of policies found that most national policies recommended the continuation or adaptation of essential health services. First, 59% (117/198) of policies recommended adapting the delivery of at least one essential health service to ensure continued provision. Second, 58% (115/198) of policies recommended continuation of at least one essential health service with infection prevention control measures. Third, 11% (22/198) of policies recommended pausing or suspending at least one essential health service.

We also performed content analysis to identify service delivery adaptations recommended in policies in five countries (Burkina Faso, Ethiopia, India, Kenya and Nigeria), revealing 16 adaptations that were designed to reduce patient load in health facilities, limit the number of visits to health facilities, limit face-to-face contact, ensure uninterrupted access to medication and deliver health services equitably. Table 1 displays the 11 most common adaptations and their frequency across health areas.

**Table 1:** Frequency of adaptations listed in policies, Burkina Faso, Ethiopia, India, Kenya and Nigeria, 2020

Adaptation	Health area^a^														
	Maternal and newborn	Noncommunicable diseases	Nutrition	Child and adolescent	HIV	Immunization	Mental disorders	Reproductive	Older people^b^	Gender-based violence	Tuberculosis	Adolescent	Neglected tropical diseases	Malaria	Total
**Integration at point of care**	8	5	5	9	8	6	2	4	2	1	2	1	1	2	56
**Appointment scheduling**	10	3	4	9	10	7	2	5	1	0	0	1	1	2	55
**Telehealth consultations**	10	4	5	8	5	2	2	6	2	0	1	2	1	1	49
**Extended service hours**	7	2	4	8	9	6	1	3	0	1	2	0	1	2	46
**Home or community-based delivery of medicines**	4	2	6	6	7	4	1	4	1	0	2	1	2	2	42
**Reconfiguration and triage of patient intake**	9	5	2	9	5	3	0	3	2	0	1	0	1	2	42
**Home-based visits**	7	1	4	6	6	5	1	2	0	1	1	1	1	2	38
**Multimonth prescribing and dosing**	4	4	5	5	7	2	0	4	0	0	2	1	2	2	38
**Intensified community outreach to generate demand**	5	1	3	10	6	5	0	1	1	1	1	0	1	1	36
**Task shifting to lower levels of care**	5	0	4	8	5	6	0	3	1	1	1	0	1	1	36
**Self-care and self-management of health**	1	2	0	2	2	0	1	2	0	0	0	1	0	0	11

^a^ Health areas as provided in the World Health Organization operational guidance on maintaining essential health services during the coronavirus disease 2019 pandemic.^5^

^b^ Older people are defined as those older than 60 years.

## Policy development fragmentation

Our review of 198 policies from 53 countries highlighted that each health area (such as maternal health, immunization) tended to have its own policy development process, resulting in an overall proliferation of policies and variable levels of attention across health areas. On average, a country had four policies available, with up to 22 documents reviewed in one country. Maternal, newborn and child health services appeared most frequently, with noncommunicable diseases coming second. Conversely, health areas such as reproductive health, adolescent health, health of people older than 60 years and tuberculosis received little policy attention. We observed that most countries adhered closely to available global guidance, and the initial development of such guidance by multiple global technical agencies and departments likely contributed to the resulting number of national policies.

The proliferation of policies within a single country complicates implementation by weakening the coherence and clarity of the policies for implementers, such as health-care workers.^7^ We found that policy fragmentation at times led to competing guidance for how specific activities should be adapted and maintained.

## Policy implementation challenges

We do not know what the results of these policies to maintain essential health services have been. Policy implementation has been hampered by lack of operational or implementation details: for example, only 45% (89/198) of policies included guidance on managing the health workforce to maintain essential health services during COVID-19, and 11% (22/198) of policies included guidance on financing the proposed changes. While our research did not include analysis of the policy process, we found no mention in the documents of engagement of implementers or health systems users in the process, further weakening the relevance and ability to implement the statutes.

The example of telemedicine offers a case study of policy implementation during the COVID-19 pandemic. Although most national policies recommended telemedicine to maintain antenatal care, birth preparedness, postnatal care and breastfeeding support during COVID-19, only 1% (1/101) of surveyed maternal and newborn health providers from low-income countries began telemedicine during the pandemic.^8^ Reasons include low awareness of the recommendations, lack of training and equipment, and provider and client concerns about quality of care.

## Lessons learnt

In the face of significant service disruptions during the COVID-19 pandemic, governments were swift in responding with COVID-19 policy guidance defining adaptations to support maintenance of essential health services. However, as the pandemic extends into a second year, little has changed; our analysis suggests further adaptations to policies after their initial release in mid-2020 were limited, and so were additional efforts to engage implementers or beneficiaries. Based on our experience from the COVID-19 policy analysis, we recommend two key learning actions to advance current recovery efforts and respond to future outbreaks.

First, future policy development efforts must aim to integrate across essential health service areas, an important step towards integrated, person-centred primary health care. Some countries were able to achieve greater policy integration including through processes such as multisectoral COVID-19 task forces or institutions (for example political commitment and movement towards primary health care and essential health services); these should be strengthened in all countries. Global technical and normative agencies can help by developing integrated guidance to ensure coherence and consistency. As countries move forward with updating primary health-care policies and operational guidance based on WHO’s Operational Framework for Primary Health Care,^9^ now is a good opportunity to consider whether and how the COVID-19-related adaptations can be incorporated into standards of care to strengthen primary health care – and to carefully consider streamlining policy adaptations within the umbrella of such care to help limit the over-proliferation of disease-specific policy adaptations and guidance.

Second, while policy development is a necessary first step, our review highlighted gaps in effective implementation. Monitoring and evaluating policy implementation and effectiveness of adaptations to deliver essential health services in a pandemic context is needed. To enable timely, evidence-informed decision-making, we recommend increased investment in rapid policy evaluation or implementation research to document which of the policy adaptations are indeed implemented, and to assess the feasibility and effectiveness of those policy adaptations in low- and middle-income countries. Many of the essential health service adaptations identified in the COVID-19 policy guidance serve as promising approaches for strengthening person-centred primary health care in a non-pandemic context, for example expanding delivery models that increase service integration or extending service hours and locations to be more responsive to user needs and preferences. Learning about which of these could strengthen essential health services and primary health care in a non-pandemic context, and how to institutionalize local innovation and adaptation is critical.

Nearly 2 years into the COVID-19 pandemic, the resilience of health systems remains threatened, as does access to and effective coverage of high-quality health care. Health policy presents a lever for change if policies reflect local needs and context, are developed through citizen engagement and are robustly monitored and adapted based on learning.
